# A LONGITUDINAL STUDY OF MEXICAN AMERICAN CAREGIVER DEPRESSIVE SYMPTOMS OVER FIVE YEARS

**DOI:** 10.1093/geroni/igae098.1569

**Published:** 2024-12-31

**Authors:** Sunshine Rote

**Affiliations:** University of Louisville, Louisville, Kentucky, United States

## Abstract

The use of different domains of informal support (e.g., neighborhood and family support) as a protective factor for Latino caregiver mental health has received little attention. The purpose of this study is to examine the role of caregiver background (that is, caregiver and care recipient relationship type, language usage), living arrangements, and family and neighborhood support for depressive symptoms among Mexican American caregivers over five years of data. Using two waves of the Hispanic Established Epidemiologic Study of the Elderly Caregiver Supplement (H-EPESE CG, 2010/2011-2016, N=333) and informed by the sociocultural caregiver stress process model, logistic and OLS regression analyses of caregiver depressive symptoms were specified. While depressive symptoms were relatively low at both waves, there was a greater increase in depressive symptoms among caregivers who completed the interview in Spanish (n= 229, 69%) compared to those completing it in English (n= 104, 31%). This increase was partially explained by lower caregiver resources (e.g., support from others). The findings demonstrate the need for dementia care supports for Mexican American caregivers, improving support systems for Spanish-speaking caregivers.

